# Description of the Dunning-Kruger effect in general surgery residents during laparoscopic cholecystectomy: a blinded prospective study

**DOI:** 10.1590/acb393224

**Published:** 2024-07-01

**Authors:** Breno Cordeiro Porto, Rodrigo Marcus Cunha Frati, Rafael Guisalberte Maltez, Amanda Ferreira da Silva Lima, Tatiane Alves Ferreira, Larissa Cunha Baron, Carlo Camargo Passerotti, Everson Luiz Artifon, José Pinhata Otoch, Jose Arnaldo Shiomi da Cruz

**Affiliations:** 1 Universidade de São Paulo – Faculdade de Medicina – Surgical Technique & Experimental Surgery –São Paulo (SP), Brazil.; 2 Universidade Nove de Julho – School of Medicine – São Paulo (SP), Brazil.; 3 Hospital Alemão Oswaldo Cruz – Urology Department – São Paulo (SP), Brazil.

**Keywords:** Cholecystectomy, Laparoscopic, Motor Skills, Education

## Abstract

**Purpose:**

The purpose of this study is to assess whether the Dunning-Kruger effect occurs in surgical residents when performing laparoscopic cholecystectomy in a porcine model.

**Methods:**

Prospective blinded study, which counted with forty PGY-1 general surgery residents who agreed to participate in the study were blindly recruited to perform a laparoscopic cholecystectomy in a porcine model. At the end of the procedure, the participants assigned a score of 0-10 for their own performance and the video of the operation was independently assessed by 2 experienced laparoscopic surgeons using a validated tool.

**Results:**

Participants were divided into groups of 10 individuals according to objective performance and compared. The group with the worst objective result was inferior to the group with the best objective result (3.77 ± 0.44 vs. 8.1 ± 0.44, p < 0.001), but they were similar in self-perception of performance (5.11 ± 1.69 vs. 6.1 ± 1.79, p = 0.999).

**Conclusions:**

In the studied sample, it was possible to demonstrate the presence of the Dunning-Kruger effect.

## Introduction

When individuals are not proficient in the strategies, they use to achieve success in a task, they face a dual challenge: they not only reach inaccurate conclusions but also, their ineptitude hinders their ability to recognize those misjudgments. As Charles Darwin insightfully noted over a century ago, “Ignorance more frequently begets confidence than does knowledge”. In essence, the very skills that lead to competence in a domain are the same ones required to evaluate competence in that domain. Consequently, underperformers often lack what cognitive psychologists term metacognition^1^ or metacomprehension^2^.

Research across various disciplines supports the idea that underachieving individuals often lack the necessary metacognitive skills for accurate self-assessment. In the realm of physics, novices are less adept than experts at gauging the complexity of problems. In tennis, beginners are less capable than seasoned players of accurately evaluating the success of their plays or their overall performance^3^^,^ 4.

Initially, Kruger and Dunning conducted four parallel experiments across different fields of knowledge. The central hypothesis across these studies remained consistent: a test was administered to participants with varying expertise in the subject, and while their scores were documented, they were also asked to estimate their performance. For instance, in one of the experiments, 65 college students were tasked with assessing the humor in jokes from renowned comedians. The students´ evaluations were then compared with those of eight professional comedians. It was found that the lowest-scoring participants overestimated their ability to discern humor. Similar results were observed in subsequent experiments across various knowledge domains. This phenomenon was coined the “Dunning-Kruger effect”^5^.

A review of prominent databases, including PubMed, Embase, and Web of Science, in October 2023, identified only three studies addressing the Dunning-Kruger effect within the context of surgery. While all these studies discussed the potential risk of bias arising from the effect, none conclusively demonstrated its existence. Theoretically, given their extensive education and training, surgeons should be less susceptible to this bias^6^^-^ 8.

Furthermore, it has been suggested by Kruger and Dunning’s subsequent research that extensive study and knowledge tend to diminish the Dunning-Kruger effect (DK effect). Performing surgeries entails considerable responsibility and risk^5^. It is deeply concerning to think that a surgeon might operate under the misconception of competence while lacking actual proficiency. Yet, no study was published so far investigating the presence of the DK effect among practicing surgeons or surgical residents. Thus, the purpose of this study is to assess the existence of the DK effect among first-year general surgery residents as they perform laparoscopic cholecystectomies on porcine models.

## Methods

### Type of study and research participants

This prospective, quantitative analytical study was conducted between 2020 and 2023, and involved 40 first-year General Surgery residents from a university hospital. Participants, aged 25 to 35, had minimal prior video laparoscopic surgical experience. Prior to the study, all residents completed an Informed Consent Form, though they remained “blinded” to the specific evaluations during the procedures. This study received prior approval from the Institutional Review Board.

Participants were selected to perform laparoscopic cholecystectomy on a porcine model under the guidance of an experienced laparoscopic surgeon. The entirety of these procedures was recorded. Once completed, each participant rated its own performance on a scale from 0 to 10. For self-assessment, a numeric scale from 0-10 was used. Zero was the worst possible performance and 10 was the best performance possible, for those who accomplished the procedure with proficiency. Following this, two laparoscopy-experienced surgeons reviewed and scored the recorded procedures using a validated tool^9^. The same numeric scale the participants used for self-assessment were used by the experienced surgeons assessing the videos. As this is not a validated scale, GOALS was also used to assess participants’ performance. All videos were assessed by both surgeons, and the objective score was the simple mean of both surgeons scores. Both raters are experienced laparoscopic surgeons from university hospitals engaged in residents’ training. Interrater reliability was not calculated between the assessments. The final performance evaluation was derived from the average of these two assessments.

### Porcine model

The procedures utilized a porcine model. The animals, weighing between 10 and 15 kg, were sourced from a farm specializing in animals for experimentation. Each animal was anesthetized using a combination of telazol (4.4-6.6 mg/kg intramuscularly), xylazine (1.1-2.2 mg/kg intramuscularly), and atropine (0.04 mg/kg intramuscularly), with anesthesia maintained using isoflurane (1-4%) mixed with 1 to 2 L of oxygen. Throughout the procedure, the animals remained intubated and on mechanical ventilation. Upon procedure completion, the animals were euthanized via an intravenous potassium chloride (KCl) injection and later incinerated as biological material in line with national animal experimentation guidelines^10^.

### Laparoscopic cholecystectomy

Laparoscopic cholecystectomy was performed with the animal in the supine position under general anesthesia. A 4-port technique was used. The experienced surgeon wielded the camera and the gallbladder with a grasper through the more lateral 5-mm trocar. Initially, the participant should dissect the pedicle of the gallbladder without separating cystic artery from the cystic duct, since these structures are closely related and technically difficult to separate in the porcine model, when compared to the human model.

After isolation of the pedicle of the gallbladder, the participant should clip the pedicle with 2 clips proximally and 1 distally to the point at which section of the pedicle should be made. The next step was to make a section of the pedicle and to free the gallbladder from its bed. Once the gallbladder was loose, the procedure was considered concluded. The blood loss was then aspirated and quantified. The entire operation was meticulously documented, and upon completion, participants self-rated their performance.

### Procedures assessment

All surgical performances were reviewed through the video recordings, focusing on both quantitative and qualitative metrics. Quantitative aspects included procedure timing for individual steps and the overall duration. Qualitative evaluation, devoid of surgeon identification, was conducted by two seasoned laparoscopic surgeons, utilizing the global operative performance scale by Vassiliou et al. This scale encompasses five facets of minimally invasive procedures and offers a score between 1 and 5 for each, leading to a potential total ranging between 5 and 25 points (Table 1)^9^. Table 1 is similar to that of Vassiliou et al.’s manuscript. Two and 4 are intermediate values and are not explicit in the table just as in the original GOALS manuscript.

**Table 1:** Global assessment scale: criteria for qualitative assessment of surgical skill^11^.

Qualitative assessment: global assessment scale	
Sense of depth	Tissue handling
1. Often overshoots target, wide swings, slow to correct 2. Sometimes overshoots or misses the target, but is quick to correct 3. Directly points the instrument at the correct plane of the target	1. Sudden movements, damage to the tissue and neighboring structures, poor control of the grasper and its fixation 2. Handles tissue reasonably well, resulting in less injury 2. Handles tissue well, applies appropriate traction, does not cause damage to neighboring structures
**Bimanual dexterity**	**Autonomy**
1. Uses only one hand, ignores non-dominant hand, poor coordination between both hands 2. Uses both hands, but does not optimize interaction between them 3. Expertly uses both hands to provide optimized exposure	1. Unable to complete the task even with verbal instructions 2. Able to complete task safely with moderate instruction 3. Able to complete task independent of instructions
**Efficiency**	**Total Score**
1. Inefficient efforts, too many attempts at movement, constantly changing approach or insisting in the same approach without making any progress 2. Slow, but planned movements are rationally organized 3. Efficient and secure, maintains focus until another approach provides better performance	5 - 25 / 25

A comprehensive assessment of all surgical procedures was conducted by reviewing video recordings, taking into account both quantitative and qualitative measures. Thus, the communication between participant and surgeon could be listened and assessed. If the participant did not need verbal instructions during the procedure, his autonomy was 5, according to GOALS. If all the procedure had to be directed by the surgeon, his autonomy was 0.

### Statistical analysis

Data derived from video analyses were compiled into spreadsheets. Qualitative variables were presented in percentages, and quantitative variables were described with mean and standard deviation. As in the initial experiments uncovering DK effect, participants performed cholecystectomies into groups of ten, according to their objective performance assessed by laparoscopy experts.

Differences in performance across these quartiles were assessed using the Kruskal-Wallis test. In instances of statistical significance, pairwise method comparisons between quartiles were employed to discern distinctive quartiles. Statistical examinations were carried out with a 5% significance threshold using IBM SPSS Statistics for Windows (Version 29.0. Armonk, NY: IBM Corp.).

## Results

The overall performance of the participants is shown in Table 2. When comparing interquartile performance, there was no difference between dominant hand (p = 0.799), age (p = 0.585), self-perception score (p = 0.394), time to ligate the gallbladder pedicle (p = 0.240) and time to section the gallbladder pedicle (p = 0.883). On the other hand, the objective score was clearly different (p < 0.001). This outcome was anticipated since grouping was based on this metric. In the quantitative assessment, the time for pedicle dissection (p = 0.023), the time for gallbladder removal (p = 0.02) and the total operative time (p = 0.021) showed statistically significant differences. In the qualitative analysis, all variables showed a statistically significant difference (p < 0.001) (Table 3).

**Table 2:** Overall performance. Data presented as percentage and mean (± standard deviation).

Overall performance	n (%)
Right-handed/left-handed	35 (87.5%) / 5 (12.5%)
Age (years)	25.2 (± 1.43)
Self-perception score	5.35 (± 1.67)
Objective score	6 (± 1.88)
**Quantitative assessment**	**Time (s)**
Dissection	410.9 (± 316)
Pedicle ligature	375.2 (± 203)
Pedicle section	64.13 (± 50.2)
Gallbladder removal	746.18 (± 470)
Total operative	1596.5 (± 621)
**Qualitative assessment**	**n (%)**
Depth perception	2.59 (± 0.72)
Bimanual dexterity	2.97 (± 0.95)
Efficiency	2.75 (± 0.89)
Tissue management	2.97 (± 1.14)
Autonomy	4.43 (± 0.76)
Overall score	15.72 (± 3.77)

**Table 3:** Performance by quartile. Data were presented as mean (± standard deviation).

	Quartile 1 (worst result)	Quartile 2	Quartile 3	Quartile 4 (best result)	p-value
Number of participants	10	10	10	10	
Right-handed/left-handed	10/0	8/2	10/0	10/0	0.799
Age (years)	25 (± 0.7)	24.7 (± 0.86)	25.3 (± 2)	25.7 (± 1.76)	0.585
Self-perception score	5.11 (± 1.69)	4.88 (± 1.45)	5.22 (± 1.71)	6.1 (± 1.79)	0.394
Objective score	3.77 (± 0.44)	5.22 (± 0.44)	6.66 (± 0.43)	8.1 (± 0.45)	**< 0.001**
**Quantitative assessment (seconds)**					
Dissection Time	662.3 (± 314.8)	400.7 (± 246.26)	303.3 (± 342.4)	290.7 (± 2 53.69)	**0.022**
Pedicle ligature time	480.7 (± 231.5)	228 (± 175.05)	399.8 (± 187.8)	291.8 (± 197)	0.240
Pedicle section time	64.1 (± 48.9)	56.2 (± 48.2)	75.4 (± 58.6)	61.1 (±51.1)	0.882
Gallbladder removal time	597.7 (± 273.4)	926.2 (± 281)	1070.7 (± 725.8)	425.6 (± 78.36)	**0.002**
Total operative time	1805 (± 465)	1721.2 (± 288.4)	1849 (± 807.8)	1069.2 (± 451.4)	**0.021**
**Qualitative assessment**					
Depth	2 (± 0.5)	2.1 (± 0.6)	2.88 (± 0.3)	3.3 (± 0.48)	**< 0.001**
Bimanual dexterity	1.88 (± 0.33)	2.55 (± 0.72)	3.33 (± 0.70)	4 (± 0)	**< 0.001**
Efficiency	1.66 (± 0.5)	2.55 (± 0.52)	2.88 (± 0.33)	3.8 (± 0.42)	**< 0.001**
Tissue management	1.88 (± 0.92)	2.44 (± 1.0)	3.44 (± 0.52)	4 (± 0.66)	**< 0.001**
Autonomy	3.44 (± 0.52)	4.44 (± 0.72)	4.77 (± 0.44)	5 (± 0)	**< 0.001**
Overall score	10.8 (± 1.69)	14.1(± 1.82)	17.3 (± 1.11)	20 (± 0.73)	**< 0.001**

## Comparison between quartiles

In line with the Dunning-Kruger effect study our primary focus was the comparison between quartiles 1 and 4. The Pairwise quartile method was applied in this case. Given multiple quartile comparisons, Bonferroni correction was applied to adjust the test significance value, thus reducing type I error likelihood.

**Figure 1:**
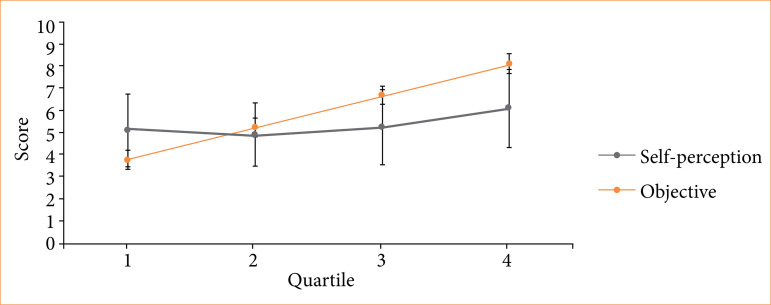
Comparison of the behavior of objective and self-perceived scores between quartiles. Quartile one overestimated its performance, while the other quartiles underestimated its performance. The better the performance, the more it was underestimated.

Comparing quartiles 1 and 4 yielded results (Table 1) similar to those of Kruger and Dunning. Notably, while self-perception showed no differences between these quartiles, objective performance and the majority of the other studied variables did (Fig. 1).

## Discussion

This experiment obtained very similar findings to that of Kruger et. al^5^, which allows us to infer that, in fact, the DK effect exists among residents during their first laparoscopic procedures. At our institution, University of Sao Paulo Faculty of Medicine, there are 24 vacancies annually for general surgery residency, with a competition of around 10 to15 candidates per vacancy, therefore, only the best 5 to -10% of candidates are admitted to the residency program. The selection process is expected to admit individuals with considerable minimum knowledge and some degree of metacognition. Despite this, the curves obtained in the experiments are similar to the initial manuscript by Kruger et. al.^11^.

The research by Kruger et. al.^11^ raises two questions. First, is there a DK effect? Second, why does it happen? Most studies recognize that there is a DK effect and provide a psychological explanation, either agreeing or disagreeing with the metacognitive explanations of Kruger and Dunning^12^^-^ 17. Theories try to explain the DK effect:

- **Metacognitive deficits:** The metacognitive hypothesis suggests that low performers don’t have the required skills to recognize their inadequacy. Supporting this idea, studies have shown that training and education can reduce the DK effect by equipping individuals with better metacognitive tools^18^.
- **Anchoring and adjustment:** Another theory posits that individuals anchor their self-assessments based on their own performance and following inadequately adjust this self-evaluation when exposed to the performance of others. Meeran et al. (2020) provided evidence for this, showing that the DK effect can be understood as an anchoring-and-adjustment phenomenon^19^.
- **Regression to the mean:** A more statistically oriented explanation, the “regression to the mean” theory suggests that the DK effect is merely a statistical artifact. Since most people believe they are above average (a mathematical impossibility), this false self-assessment leads to an apparent overestimation of abilities among low performers and an underestimation among high performers^20^.
- **Motivated reasoning:** DK effect arises from an innate human desire to view oneself in a positive light. This “self-serving bias” could lead to overestimations of one’s abilities to maintain self-esteem^21^.

While the existence of the Dunning-Kruger Effect is broadly supported by empirical evidence, the underpinnings of its cause remain multifaceted, ranging from metacognitive shortcomings to statistical phenomena. Regardless of the reason behind this, the DK effect offers a cautionary reminder of the limits of self-awareness and the potential pitfalls of overconfidence^22^.

Our experiment certainly has limitations: the reduced number of participants could be a limiting factor, as well as the lack of similar studies in surgery to estimate the sample size. Nevertheless, by performing the post-hoc calculation of statistical power, we obtained a power above 80%, which is very positive, despite having all the inherent limitations of the post-hoc calculation of statistical power. The inputs to carry out this study are costly, both financially and ethically. As the work was performed in a public educational institution, resources were managed in the most optimized way.

Further studies should assess different groups of surgeons, such as senior residents and experienced surgeons. Furthermore, the impact that training will have on these surgeons must be assessed with the aim of optimizing their metacognition and mitigating the DK effect as much as possible in order to obtain the best results for patients.

## Conclusion

Our study effectively depicted the DK effect among general surgery residents performing laparoscopic cholecystectomies on a porcine model. Further research is essential to validate our results and develop strategies to mitigate the DK effect’s implications.

## Data Availability

Data available upon request.
